# Comparative effectiveness of first-line palbociclib plus letrozole versus letrozole alone for HR+/HER2− metastatic breast cancer in US real-world clinical practice

**DOI:** 10.1186/s13058-021-01409-8

**Published:** 2021-03-24

**Authors:** Angela DeMichele, Massimo Cristofanilli, Adam Brufsky, Xianchen Liu, Jack Mardekian, Lynn McRoy, Rachel M. Layman, Birol Emir, Mylin A. Torres, Hope S. Rugo, Richard S. Finn

**Affiliations:** 1 Abramson Cancer Center, University of Pennsylvania, 3400 Civic Center Blvd, Philadelphia, PA 19104 USA; 2 Robert H. Lurie Cancer Center of Northwestern University, Feinberg School of Medicine, Chicago, 710 N Fairbanks Ct, Suite 8-250A, Chicago, IL 60611 USA; 3 UPMC Hillman Cancer Center, University of Pittsburgh Medical Center, 300 Halket Street, Pittsburgh, PA 15213 USA; 4 Pfizer Inc, 235 42nd St, New York, NY 10017 USA; 5 The University of Texas MD Anderson Cancer Center, 1515 Holcombe Boulevard, Unit 1354, Houston, TX 77030 USA; 6 Winship Cancer Institute, Emory University School of Medicine, 1365 Clifton Rd. NE, Building A, 1st Floor, Rm. 1307A, Atlanta, GA 30322 USA; 7 University of California San Francisco Helen Diller Family Comprehensive Cancer Center, 1825 4th Street, 3rd Floor, Box 1710, San Francisco, CA 94158 USA; 8 David Geffen School of Medicine at University of California Los Angeles, 2825 Santa Monica Blvd, Suite 200, Santa Monica, CA 90404 USA

**Keywords:** HR+/HER2−, Metastatic breast cancer, Palbociclib, Letrozole, Real-world data, Comparative effectiveness

## Abstract

**Background:**

Findings from randomized clinical trials may have limited generalizability to patients treated in routine clinical practice. This study examined the effectiveness of first-line palbociclib plus letrozole versus letrozole alone on survival outcomes in patients with hormone receptor–positive (HR+)/human epidermal growth factor receptor–negative (HER2−) metastatic breast cancer (MBC) treated in routine clinical practice in the USA.

**Patients and methods:**

This was a retrospective observational analysis of electronic health records within the Flatiron Health Analytic Database. A total of 1430 patients with ≥ 3 months of follow-up received palbociclib plus letrozole or letrozole alone in the first-line setting between February 3, 2015, and February 28, 2019. Stabilized inverse probability treatment weighting (sIPTW) was used to balance baseline demographic and clinical characteristics. Real-world progression-free survival (rwPFS) and overall survival (OS) were analyzed.

**Results:**

After sIPTW adjustment, median follow-up was 24.2 months (interquartile range [IQR], 14.2–34.9) in the palbociclib group and 23.3 months (IQR, 12.7–34.3) in those taking letrozole alone. Palbociclib combination treatment was associated with significantly longer median rwPFS compared to letrozole alone (20.0 vs 11.9 months; hazard ratio [HR], 0.58; 95% CI, 0.49–0.69; *P* < 0.0001). Median OS was not reached in the palbociclib group and was 43.1 months with letrozole alone (HR, 0.66; 95% CI, 0.53–0.82; *P* = 0.0002). The 2-year OS rate was 78.3% in the palbociclib group and 68.0% with letrozole alone. A propensity score matching analysis showed similar results.

**Conclusions:**

In this “real-world” population of patients with HR+/HER2− MBC, palbociclib in combination with endocrine therapy was associated with improved survival outcomes compared with patients treated with letrozole alone in the first-line setting.

**Trial registration:**

Clinicaltrials.gov; NCT04176354

**Supplementary Information:**

The online version contains supplementary material available at 10.1186/s13058-021-01409-8.

## Background

An estimated 13% of women will be diagnosed with invasive breast cancer in their lifetime, and 3% will die from the disease [1]. Metastatic breast cancer (MBC; i.e., breast cancer that has spread to distant sites) is an incurable disease with a 5-year survival rate of 28.1% among women [2]. Current treatment guidelines recommend the addition of a cyclin-dependent kinase 4/6 (CDK4/6) inhibitor in combination with endocrine therapy for the treatment of patients with hormone receptor–positive (HR+)/human epidermal growth factor receptor 2–negative (HER2−) MBC based upon multiple prospective randomized phase III trials in the first- and second-line setting [3–5].

Palbociclib, the first-in-class CDK4/6 inhibitor, is approved for the treatment of HR+/HER2− MBC in combination with an aromatase inhibitor or fulvestrant [6]. The approval of palbociclib in the USA by the Food and Drug Administration was based on findings from 3 pivotal clinical trials: PALOMA-1 [7, 8] and PALOMA-2 [9, 10] evaluated palbociclib in combination with letrozole versus letrozole alone or placebo plus letrozole as the initial treatment for postmenopausal women in the advanced setting; PALOMA-3 [11–15] evaluated combination treatment with fulvestrant versus fulvestrant alone in premenopausal or postmenopausal women who had progressed following endocrine therapy. The final overall survival (OS) results from PALOMA-1 showed that palbociclib plus letrozole had a longer median OS than letrozole alone, although the improvement was not statistically significant (37.5 vs 34.5 months; hazard ratio, 0.90 [95% CI, 0.62–1.29]; *P* = 0.281) [8]. Similarly, although not statistically significant, palbociclib plus fulvestrant was associated with a longer median OS than placebo plus fulvestrant in PALOMA-3 (34.9 vs 28.0; hazard ratio, 0.81 [95% CI, 0.64–1.03]; *P* = 0.09), preserving the 7-month improvement in progression-free survival (PFS) [15]. The OS data for PALOMA-2 are not yet available.

Real-world data are increasingly being used to understand the safety and effectiveness of new drug regimens in actual clinical practice [16, 17]. Inherent limitations of real-world analyses using data collected during routine care include the lack of random treatment assignment, variations in follow-up and lack of uniform assessment of disease progression, missing data or erroneous data entry, and incomplete capture of comorbid conditions and performance status. Published real-world studies of palbociclib have shown an efficacy and safety profile consistent with that observed in clinical trials; however, these studies were limited by small sample sizes, the lack of a control group, and/or short durations of follow-up [18–20]. The goal of this study was to provide real-world evidence regarding the effectiveness of palbociclib plus letrozole versus letrozole alone in a large cohort of patients from across the USA in routine clinical practice.

## Methods

### Study design and data source

This retrospective analysis of electronic health records (EHRs) used de-identified patient data from the Flatiron Health Analytic Database, a longitudinal database that includes structured and unstructured EHRs from > 280 cancer clinics, including approximately 800 sites of care, and represents 2.4 million patients with cancer actively being treated in the USA. A patient attrition diagram is presented in Additional file 1. Patients included in this analysis met the following inclusion criteria: women aged ≥ 18 years at MBC diagnosis with HR+/HER2− MBC before or up to 60 days after the metastatic diagnosis date; a date of first prescription (index date) for palbociclib plus letrozole or letrozole alone as first-line therapy for MBC beginning on the date of the US Food and Drug Administration approval of palbociclib, February 3, 2015, and extending 4 years to February 28, 2019; with a minimum potential follow-up for ≥ 3 months from the index date to the study cutoff date of May 31, 2019. Patients were excluded if they received prior treatment with CDK4/6 inhibitors, aromatase inhibitors, fulvestrant, tamoxifen, raloxifene, or toremifene in the metastatic setting; had a first structured activity (a recording of vital information, a medication administration, a non-canceled drug order, or a reported laboratory test/result) > 90 days after the MBC diagnostic date; or received a CDK4/6 inhibitor as part of a clinical trial.

### Outcomes

These comparative analyses were conducted to assess real-world PFS (rwPFS) as the primary outcome and OS as the secondary outcome in patients with MBC treated with palbociclib plus letrozole versus letrozole alone as first-line therapy [21, 22]. rwPFS was defined as the time in months from the start of palbociclib plus letrozole or letrozole alone therapy to death or disease progression. Disease progression was determined by the recorded assessment of the treating clinician based on radiology, pathology, clinical assessment, or laboratory evidence. Patients who did not die or have disease progression were censored at the date of initiation of the next line of therapy for those with ≥ 2 lines of therapy or at their last visit during the study period of February 2015 to May 2019 for patients with only 1 line of therapy. The duration of follow-up was defined as the time in months from the start of palbociclib plus letrozole or letrozole alone to death or the data cutoff date of May 31, 2019, whichever came first.

Overall survival was defined as the number of months from the start of treatment with palbociclib plus letrozole or letrozole alone to death due to any cause as recorded by Flatiron in the data extract. The date of death was acquired from a recent mortality dataset generated by combining multiple data sources and benchmarked against the National Death Index [23]. Patients who did not die were censored at the study cutoff date (May 31, 2019).

### Statistical analyses

Means, standard deviations, medians, and interquartile ranges (IQRs) were calculated for continuous variables. Counts and percentages were reported for dichotomous and polychotomous variables. *T*-tests or chi-square tests, as appropriate, were performed to compare treatment groups for baseline demographic and clinical characteristics. The Kaplan-Meier method and 95% CIs were used to estimate medians for rwPFS and OS. Cox proportional regression analyses with a robust sandwich estimator were used to estimate hazard ratios and 95% CIs for rwPFS and OS events. In subgroup analyses, a subgroup by treatment interaction term was included.

Comparative analyses were conducted and presented by 3 methods: unadjusted (without controlling for confounders), stabilized inverse probability treatment weighting (sIPTW) method (the primary analysis that controlled for observed confounders), and finally the propensity score matching (PSM) method (a sensitivity analysis that assessed the robustness of the primary analysis results). Propensity score calculation and its application as either matching (PSM) or weights (sIPTW) were conducted to balance baseline demographic and clinical characteristics and to adjust for differences in observed potential confounders between the two cohorts. The sIPTW approach was used as the primary analysis. A multivariable binomial logistic regression model was used to generate the propensity scores [24–27]. Patients in the two study cohorts were matched by the propensity score matching (PSM) method. The nearest neighbor method (without replacement and with a caliper of 0.01 [24]) was used to select the matched samples. PSM of baseline demographic and clinical characteristics at a ratio of 1:1 then identified 464 patients in each treatment group. This approach was used as a sensitivity analysis to determine the robustness of the findings from the primary analyses [28].

To mitigate the effect of missing survival data, a major confounding factor in real-world oncology analyses, and in the absence of reliable information on the cause of missing data, calculation of percentages always included a “missing” category presented by treatment. For some categorical variables, “missing” was included as another level, e.g., Eastern Cooperative Oncology Group (ECOG) performance status included a level of “not documented.”

## Results

### Patients

A total of 1430 women with HR+/HER2− MBC who started palbociclib plus letrozole (*n* = 772) or letrozole alone (*n* = 658) as first-line therapy between February 3, 2015, and February 28, 2019, were identified from the Flatiron Database. Most patients (94.0%) were from a community setting; 6.0% of patients were from an academic setting. Demographic and clinical characteristics differed between the palbociclib plus letrozole and letrozole alone groups. Compared with the letrozole group, patients in the palbociclib plus letrozole cohort were younger and had better performance status, a higher incidence of visceral disease, and a greater number of metastatic sites (Table 1). Patient characteristics were generally balanced after sIPTW adjustment (Table 1) and between propensity score–matched cohorts (Additional file 2). After sIPTW adjustment, the mean (SD) age was 66.8 (11.2) years in the palbociclib group and 67.1 (11.1) years in the letrozole group, and approximately 68.0% of patients in each group were white. The median follow-up duration was 24.2 months (IQR, 14.2–34.9) for patients who received palbociclib plus letrozole and 23.3 months (IQR, 12.7–34.3) for patients who received letrozole alone.

**Table 1:** Patient characteristics

	Unadjusted total cohort			Cohort after sIPTW		
Characteristic	Palbociclib + letrozole (*n* = 772)	Letrozole (*n* = 658)	Standardized difference	Palbociclib + letrozole (*n* = 839)	Letrozole (*n* = 698)	Standardized difference
Age, y						
Mean (SD)	65.2 (10.4)	69.2 (10.9)	− 0.3793	66.8 (11.2)	67.1 (11.1)	− 0.0288
Median (IQR)	66.0 (58.0–73.0)	70.0 (61.0–79.0)		67.0 (59.0–75.0)	68.0 (60.0–76.0)	
Age group*, *n* (%), y						
18–49	52 (6.7)	30 (4.6)	0.0944	48 (5.8)	38 (5.5)	0.0114
50–64	304 (39.4)	187 (28.4)	0.2331	288 (34.3)	244 (35.0)	− 0.0131
65–74	262 (33.9)	193 (29.3)	0.0992	265 (31.6)	221 (31.7)	− 0.0025
≥ 75	154 (19.9)	248 (37.7)	− 0.3995	237 (28.3)	194 (27.8)	0.0107
Race/ethnicity*, *n* (%)						
White	525 (68.0)	446 (67.8)	0.0048	572 (68.1)	470 (67.4)	0.0163
Black	54 (7.0)	60 (9.1)	− 0.0781	62 (7.4)	55 (7.9)	− 0.0187
Asian	13 (1.7)	10 (1.5)	0.0131	15 (1.7)	11 (1.5)	0.0180
Hispanic or Latino	22 (2.8)	15 (2.3)	0.0361	23 (2.7)	16 (2.3)	0.0264
Not documented^†^	158 (20.5)	127 (19.3)	0.0292	167 (20.0)	146 (20.9)	− 0.0225
Practice type*, *n* (%)						
Academic	46 (6.0)	31 (4.7)	0.0555	44 (5.2)	36 (5.2)	0.0010
Community	726 (94.0)	627 (95.3)		795 (94.8)	661 (94.8)	
Disease stage at initial diagnosis*, *n* (%)						
I	82 (10.6)	79 (12.0)	− 0.0437	90 (10.8)	78 (11.2)	− 0.0127
II	187 (24.2)	149 (22.6)	0.0373	198 (23.6)	161 (23.1)	0.0129
III	109 (14.1)	97 (14.7)	− 0.0177	121 (14.4)	101 (14.4)	− 0.0017
IV	321 (41.6)	254 (38.6)	0.0608	338 (40.3)	283 (40.6)	− 0.0062
Not documented	73 (9.5)	79 (12.0)	− 0.0825	91 (10.9)	74 (10.7)	0.0069
ECOG PS*, *n* (%)						
0	293 (38.0)	167 (25.4)	0.2728	271 (32.3)	227 (32.5)	− 0.0059
1	159 (20.6)	134 (20.4)	0.0057	175 (20.9)	144 (20.7)	0.0055
2, 3, or 4	49 (6.3)	84 (12.8)	− 0.2196	82 (9.7)	66 (9.5)	0.0095
Not documented	271 (35.1)	273 (41.5)	− 0.1316	311 (37.1)	260 (37.3)	− 0.0047
Visceral disease*^,‡^, *n* (%)						
No	442 (57.3)	437 (66.4)		518 (61.8)	429 (61.5)	
Yes	330 (42.7)	221 (33.6)	0.1894	320 (38.2)	269 (38.5)	− 0.0060
Bone-only disease*^,§^, *n* (%)						
No	501 (64.9)	398 (60.5)		530 (63.1)	440 (63.1)	
Yes	271 (35.1)	260 (39.5)	− 0.0913	309 (36.9)	258 (36.9)	− 0.0011
Brain metastases, *n* (%)						
No	753 (97.5)	628 (95.4)		820 (97.7)	656 (94.0)	
Yes	19 (2.5)	30 (4.6)	− 0.1142	19 (2.3)	42 (6.0)	− 0.1901
Time from initial Dx to metastatic Dx*, *n* (%), y						
De novo	321 (41.6)	254 (38.6)	0.0608	338 (40.3)	283 (40.6)	− 0.0062
≤ 1	19 (2.5)	23 (3.5)	− 0.0609	25 (2.9)	21 (3.1)	− 0.0070
> 1–5	123 (15.9)	111 (16.9)	− 0.0253	133 (15.9)	111 (16.0)	− 0.0016
> 5	308 (39.9)	269 (40.9)	− 0.0201	342 (40.7)	281 (40.3)	0.0097
Not documented	1 (0.1)	1 (0.2)	− 0.0060	1 (0.1)	1 (0.1)	0.0010
Number of metastatic sites*^,ǁ^, *n* (%)						
1	372 (48.2)	365 (55.5)	− 0.1462	423 (50.4)	355 (50.9)	− 0.0104
2	220 (28.5)	147 (22.3)	0.1418	217 (25.9)	183 (26.2)	− 0.0070
3	110 (14.2)	64 (9.7)	0.1396	101 (12.0)	86 (12.4)	− 0.0104
4	40 (5.2)	18 (2.7)	0.1257	35 (4.2)	27 (3.8)	0.0184
≥ 5	20 (2.6)	10 (1.5)	0.0755	18 (2.2)	15 (2.2)	0.0002
Not documented	10 (1.3)	54 (8.2)	−0.3293	44 (5.3)	31 (4.5)	0.0371

The balance in important prognostic baseline characteristics was assessed using a standardized difference approach, with a standardized difference of ≥ 0.10 considered indicative of practical significance [24]. The total patient population for different subgroups varied due to the application of sIPTW. Therefore, the total *n* number for each subgroup may not have always equaled the *N* number of the treatment arm (due to rounding error and categorization differences). Calculated percentages were based on the number of patients reported within each subgroup

*Dx* diagnosis, *ECOG PS* Eastern Cooperative Oncology Group performance status, *IQR* interquartile range, *sIPTW* stabilized inverse probability treatment weighting

*Variable used in the propensity score matching model; de novo vs not de novo were used as categories for initial Dx to metastatic Dx

^†^Race data were not known in the “not documented” race group

^‡^Visceral disease was defined as metastatic disease in the lung and/or liver; patients could have had other sites of metastases. No visceral disease was defined as no lung or liver metastases

^§^Bone-only disease was defined as metastatic disease in the bone only

^ǁ^Multiple metastases at the same site were counted as 1 site (e.g., if a patient had 3 bone metastases in the spine, it was considered only 1 site)

### Real-world progression-free survival

**Fig. 1:**
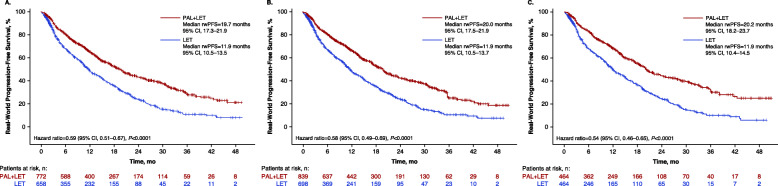
Kaplan-Meier curves of real-world progression-free survival in **a** the unadjusted analysis (number of patients at risk are shown), **b** sIPTW-adjusted analysis (number of patients at risk are shown), and **c** after PSM. LET, letrozole; NR, not reached; PAL, palbociclib; PSM, propensity score matching; rwPFS, real-world progression-free survival; sIPTW, stabilized inverse probability of treatment weighting

In the unadjusted analysis of the full cohort (*n* = 1430), median rwPFS was significantly longer among patients in the palbociclib group versus the letrozole group (*P* < 0.0001; Fig. 1a). After sIPTW adjustment, rwPFS was 20.0 months (95% CI, 17.5–21.9) among patients treated with palbociclib plus letrozole (*n* = 839) compared with 11.9 months (95% CI, 10.5–13.7) among patients treated with letrozole alone (*n* = 698; hazard ratio, 0.58 [95% CI, 0.49–0.69]; *P* < 0.0001; Fig. 1b). In a sensitivity analysis using the PSM method (*n* = 928), median rwPFS was also significantly longer among patients who received palbociclib plus letrozole (20.2 months [95% CI, 18.2–23.7] versus 11.9 months [95% CI, 10.4–14.5]; hazard ratio, 0.54 [95% CI, 0.46–0.65]; *P* < 0.0001; Fig. 1c).

**Fig. 2:**
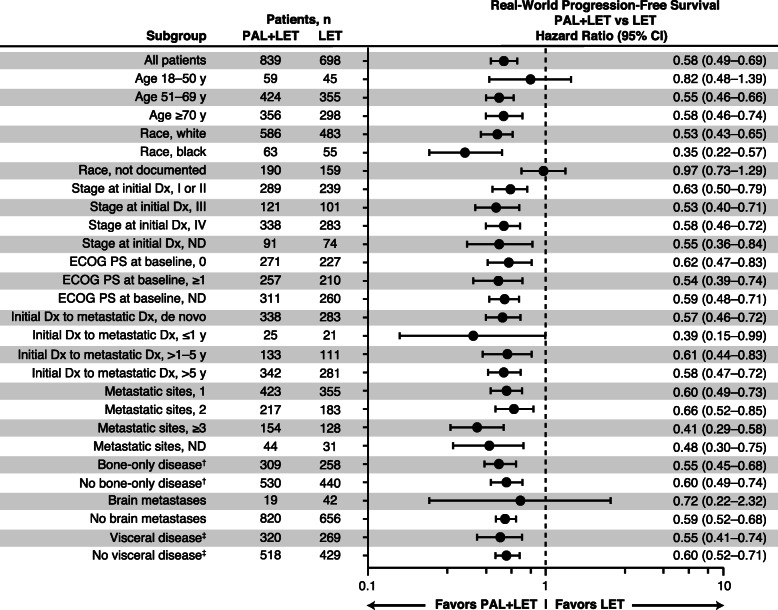
Forest plot of real-world progression-free survival by subgroup* after sIPTW. Dx, diagnosis; ECOG PS, Eastern Cooperative Oncology Group performance status; LET, letrozole; ND, not documented; PAL, palbociclib; sIPTW, stabilized inverse probability of treatment weighting. *Race by Cohort interaction was the only subgroup variable-by-treatment cohort interaction that was significant (*P*=0.0010); however, race data were not known in the “not documented” race group.^†^Bone-only disease was defined as metastatic disease in the bone only.^‡^Visceral disease was defined as metastatic disease in the lung and/or liver; patients could have had other sites of metastases. No visceral disease was defined as no lung or liver metastases. The total patient population for different subgroups varied due to the application of sIPTW. Therefore, the total *n* number for each subgroup may not have always equaled the *N* number of the treatment arm (due to rounding error and categorization differences)

A consistent PFS benefit with palbociclib plus letrozole versus letrozole alone was observed generally across all subgroups examined (Fig. 2). Notably, a similar rwPFS benefit was observed with palbociclib plus letrozole versus letrozole alone among younger patients (18–50 years, *n* = 104) and older patient groups (51–69 years, *n* = 779; ≥ 70 years, *n* = 654) and among patients with and without visceral metastases or bone-only disease. Race by cohort interaction was the only subgroup variable-by-treatment cohort interaction that was significant (*P* = 0.0010); however, race data were not known in the “not documented” race group. Similar subgroup results were also observed in the PSM-adjusted sensitivity analysis (online Additional file 3).

### Overall survival

**Fig. 3:**
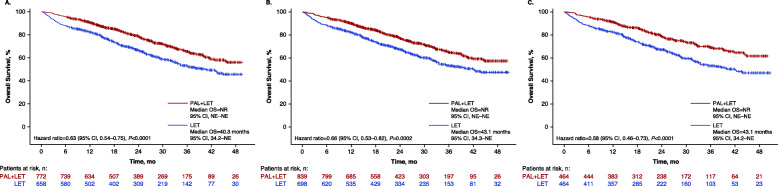
Kaplan-Meier curves of overall survival in **a** the unadjusted analysis (number of patients at risk are shown), **b** sIPTW-adjusted analysis (number of patients at risk are shown), and **c** after PSM. LET, letrozole; NE, not estimable; NR, not reached; OS, overall survival; PAL, palbociclib; PSM, propensity score matching; sIPTW, stabilized inverse probability of treatment weighting

In the unadjusted analysis, among patients who received letrozole alone, median OS was 40.3 months (95% CI, 34.2–not estimable) and was not reached in patients who received palbociclib plus letrozole (*P* < 0.0001; Fig. 3a). After sIPTW-adjusted analysis, OS was not reached (95% CI, not estimable) in patients who received palbociclib plus letrozole and was 43.1 months (95% CI, 34.3–not estimable) in patients who received letrozole alone (hazard ratio, 0.66 [95% CI, 0.53–0.82]; *P* = 0.0002; Fig. 3b). More patients died in the letrozole alone group (*n* = 266; 40.4%) compared with the palbociclib plus letrozole group (*n* = 210; 27.2%). In landmark analyses at 2 years of follow-up, the OS rate was 78.3% among patients who received palbociclib plus letrozole versus 68.0% among patients who received letrozole alone. At 3 years of follow-up, the OS rate was 64.8% in the palbociclib group and 53.2% in the letrozole group. Using PSM as a sensitivity analysis, median OS was 43.1 months (95% CI, 34.2–not estimable) in the letrozole alone group and was not reached in the palbociclib plus letrozole group (hazard ratio, 0.58 [95% CI, 0.46–0.73]; *P* < 0.0001; Fig. 3c).

**Fig. 4:**
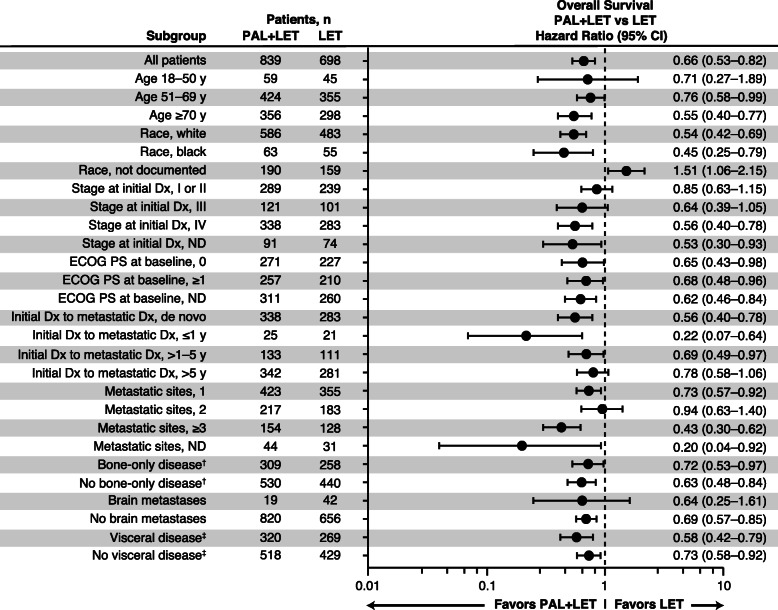
Forest plot of overall survival by subgroup* after sIPTW. Dx, diagnosis; ECOG PS, Eastern Cooperative Oncology Group performance status; LET, letrozole; ND, not documented; PAL, palbociclib; sIPTW, stabilized inverse probability of treatment weighting. *Race by Cohort interaction and Metastatic Sites by Cohort interaction were the only subgroup variable-by-treatment cohort interaction that were significant (*P*<0.0001 and *P*=0.0050, respectively); however, race data were not known in the “not documented” race group.^†^Bone-only disease was defined as metastatic disease in the bone only.^‡^Visceral disease was defined as metastatic disease in the lung and/or liver; patients could have had other sites of metastases. No visceral disease was defined as no lung or liver metastases. The total patient population for different subgroups varied due to the application of sIPTW. Therefore, the total *n* number for each subgroup may not have always equaled the *N* number of the treatment arm (due to rounding error and categorization differences)

In subgroup analyses of OS, a benefit with palbociclib plus letrozole versus letrozole alone was generally seen across the subgroups examined (Fig. 4). Consistent with rwPFS findings, a benefit of palbociclib plus letrozole versus letrozole alone on OS was observed among younger patients (18–50 years) and older patient groups (51–69 and ≥ 70 years) and among patients with and without visceral metastases or bone-only disease. Race by cohort interaction and metastatic sites by cohort interaction were the only subgroup variable-by-treatment cohort interactions that were significant (*P* < 0.0001 and *P* = 0.0050, respectively); however, data assigning race were “not documented” in approximately 23% of patients in each treatment group and findings should be interpreted with caution. Similar subgroup results were observed in the PSM-adjusted sensitivity analysis, but only race by cohort interaction had a significant subgroup variable-by-treatment cohort interaction (*P* = 0.0076; Additional file 4).

### Subsequent second-line anticancer treatments

Subsequent (second-line) treatments following first-line palbociclib plus letrozole or letrozole alone treatments are shown in Table 2. A total of 55.7% of patients (46.6% of patients in the palbociclib plus letrozole group and 66.6% of patients in the letrozole alone group) had data available on second-line treatment. Among these patients, 42.2% in the palbociclib group and 62.9% in the letrozole group received a second-line regimen with a CDK4/6 inhibitor, and 25.6% and 9.4%, respectively, received second-line chemotherapy. Outcomes related to second-line therapy were not collected as part of this study.

**Table 2:** Subsequent second-line anticancer treatments in cohorts after sIPTW analysis

Treatments, ***n*** (%)	Palbociclib + letrozole (***n*** = 839)	Letrozole alone (***n*** = 698)
First-line treatment only*	448 (53.4)	233 (33.4)
Any second-line treatment received^†^	391 (46.6)	465 (66.6)
CDK4/6 inhibitor	165 (42.2)	292 (62.9)
Chemotherapy	100 (25.6)	44 (9.4)
Endocrine therapy alone	64 (16.4)	96 (20.7)
Other anticancer treatments	88 (22.5)	56 (12.0)

*CDK4/6* cyclin-dependent kinase 4/6, *sIPTW* stabilized inverse probability of treatment weighting

*Includes patients who continued treatment, died, or were censored in the first-line setting

^†^Patients could have received > 1 category of second-line treatment

## Discussion

With only 3% of patients with breast cancer participating in randomized clinical trials [29], real-world evidence is essential to evaluate the generalizability of clinical trial findings across a heterogeneous patient population and among patients who would not have met clinical trial inclusion criteria [16]. Using electronic health records from the large multicenter Flatiron Health Analytic Database, we found that first-line palbociclib plus letrozole was associated with significantly longer rwPFS and OS than letrozole alone after sIPTW. A landmark OS analysis at 2 and 3 years of follow-up demonstrated significantly higher OS rates in the palbociclib combination group. A consistent benefit of palbociclib plus letrozole was observed across multiple subgroups. Results from the PSM sensitivity analysis also supported the results from the sIPTW primary analysis.

These findings from a large cohort of real-world patients are complementary to the clinical efficacy findings of palbociclib plus endocrine therapy observed in randomized clinical trials [7, 10, 12]. The rwPFS benefit of palbociclib plus letrozole versus letrozole alone (hazard ratio, 0.58 [95% CI, 0.49–0.69]; *P* < 0.0001) observed in the current study is consistent with PFS results from the PALOMA-2 trial (hazard ratio, 0.56 [95% CI, 0.46–0.69]; *P* < 0.0001) [9, 10]. Patients in the present (unadjusted) analysis were older than those enrolled in PALOMA-2, and a higher percentage of patients across both cohorts had bone-only disease at baseline compared with PALOMA-2. The current real-world data analysis also demonstrated a significant OS benefit with palbociclib plus letrozole versus letrozole alone (hazard ratio, 0.66 [95% CI, 0.53–0.82]). The OS benefit was generally consistent across most subgroups. However, it is unknown if the OS benefit observed in this real-world analysis would be consistent with PALOMA-2 results as OS data are not yet available.

Although there are intrinsic limitations to retrospective analyses of real-world data, the large size and geographic distribution of the Flatiron Database is a strength of this study. Real-world effectiveness endpoints for this analysis include rwPFS and OS; a recent publication by Bartlett et al. validated the real-world effectiveness endpoints as defined by Flatiron in comparison with randomized clinical trial data [30], and data from Flatiron have been used to support the label expansion for palbociclib to include men [31]. In addition, the OS endpoint from the Flatiron database includes external data sources (i.e., the National Death Index, US Social Security Death Index, obituaries, and commercial death data) in addition to health records and has been validated [23]. As described previously, the absence of randomization in treatment assignment common to observational data is an important factor that must be accounted for through appropriate statistical tools to adjust for differences in baseline and clinical characteristics. The statistical approach used in this analysis to address observed confounders included the use of PSM to balance patient demographic and clinical characteristics; PSM results were consistent with sIPTW analyses.

Unobserved variables cannot be fully addressed through sIPTW or PSM and thus may confound these findings; however, this analysis adjusted for the known clinical confounders that were most likely to have a major impact on the outcomes of the study. The 5-year survival for female patients with MBC remains < 30% [2]; thus, the impact of those potential variables on overall survival as an outcome would have to be profound to affect the findings of the current study. Some subgroups, such as younger patients (aged ≤ 50 years), may have insufficient cohort size to detect significant differences in outcomes. At the end of the study period (data cutoff), over a third of patients remained on treatment, and both cohorts had a high frequency of a CDK4/6 inhibitor-containing regimen as a subsequent second-line therapy. The use of CDK4/6 inhibitors beyond the first-line setting may have affected the overall survival results by overestimating the overall survival in the letrozole alone cohort, therefore biasing the results towards the null. However, definitive analysis of the impact of subsequent treatments on overall survival was limited by the small percentage of patients with available data. The efficacy of subsequent CDK4/6 inhibitor treatments beyond initial disease progression is an active area of investigation, and additional studies, including analysis of rwPFS after subsequent therapy in real-world clinical practice, are needed to understand the impact of treatment sequencing. Disease progression was based on the individual treating physician’s clinical assessment or interpretation of radiographic or pathologic results and not on standard criteria, such as Response Evaluation Criteria in Solid Tumors. Additionally, findings from the Flatiron Database may not be generalized to other patient populations. Finally, although median OS was reached in the letrozole alone group, significant censoring in the OS analysis highlights the need for subsequent evaluation with longer follow-up.

## Conclusions

Although findings from the PALOMA trials demonstrate the efficacy of palbociclib among patients who met study inclusion and exclusion criteria, real-world evidence of effectiveness provides further support for clinical decision-making in patients encountered in routine clinical practice [16, 32]. In this comparative effectiveness analysis, first-line palbociclib plus letrozole was associated with longer rwPFS and OS than letrozole alone in a heterogeneous population and among various patient subgroups. While overall survival data are not yet available for the PALOMA-2 randomized controlled trial, these results expand our understanding of clinical outcomes in the real-world setting and provide additional evidence regarding the use of palbociclib in combination with an aromatase inhibitor as a first-line treatment of patients with HR+/HER2− MBC.

## Supplementary Information

**Additional file 1.** Patient Attrition Diagram.

**Additional file 2.** Patient Characteristics After PSM.

**Additional file 3.** Forest Plot of Real-World Progression-Free Survival by Subgroup After PSM*.

**Additional file 4.** Forest Plot of Overall Survival by Subgroup* After PSM.

## Data Availability

Upon request, and subject to certain criteria, conditions, and exceptions (see https://www.pfizer.com/science/clinical-trials/trial-data-and-results for more information), Pfizer will provide access to individual de-identified participant data from Pfizer-sponsored global interventional clinical studies conducted for medicines, vaccines, and medical devices (1) for indications that have been approved in the USA and/or EU or (2) in programs that have been terminated (i.e., development for all indications has been discontinued). Pfizer will also consider requests for the protocol, data dictionary, and statistical analysis plan. Data may be requested from Pfizer trials 24 months after study completion. The de-identified participant data will be made available to researchers whose proposals meet the research criteria and other conditions, and for which an exception does not apply, via a secure portal. To gain access, data requestors must enter into a data access agreement with Pfizer.

## Abbreviations

CDK4/6: Cyclin-dependent kinase 4/6
ECOG: Eastern Cooperative Oncology Group
EHR: Electronic health record
HR+: Hormone receptor–positive
HER2−: Human epidermal growth factor receptor 2–negative
IQR: Interquartile range
MBC: Metastatic breast cancer
OS: Overall survival
PFS: Progression-free survival
PSM: Propensity score matching
rwPFS: Real-world PFS
sIPTW: Stabilized inverse probability treatment weighting
