# Supplementary material for: Comparative effectiveness of first-line palbociclib plus letrozole versus letrozole alone for HR+/HER2− metastatic breast cancer in US real-world clinical practice
Source: Breast Cancer Res. 2021 Mar 24;23:37. doi: 10.1186/s13058-021-01409-8 (PMC7989035; doi:10.1186/s13058-021-01409-8)
Supplement: Supplementary file 1 — Additional file 1. Patient Attrition Diagram. [file 13058_2021_1409_MOESM1_ESM.docx]

**Additional File 1.** Patient Attrition Diagram


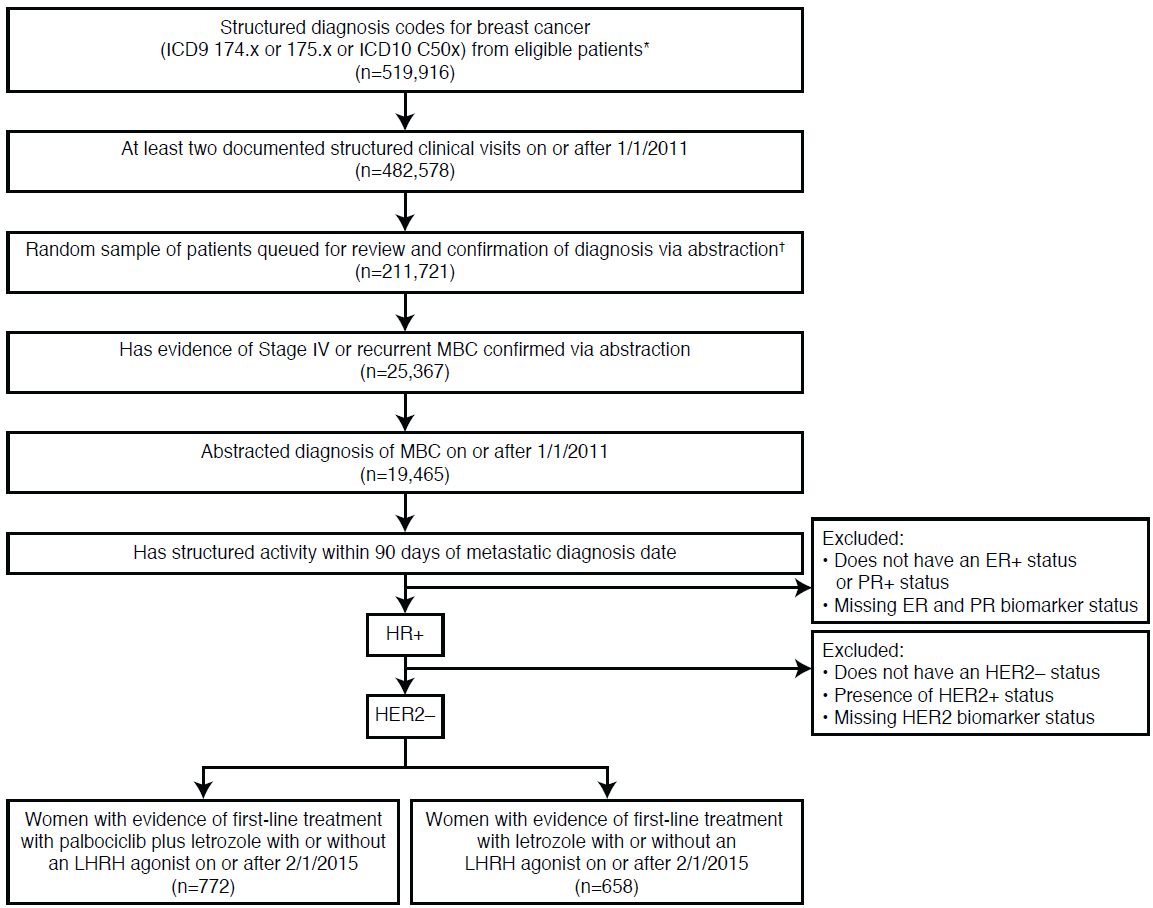


ER=estrogen receptor; HR=hormone receptor; HER2=human epidermal growth factor receptor 2; LHRH=luteinizing hormone-releasing hormone; MBC=metastatic breast cancer; PR=progesterone receptor.

*A select number of practices are excluded from this study due to known documentation limitations.

^†^Flatiron’s proprietary cohort selection methodology combines structured and unstructured data to ensure selection of patients with the diagnosis of interest. Patients are initially selected into a broad cohort based on ICD-9 or ICD-10 codes for the disease of interest, but are selected into the final analytic cohort using a technique called Model-Associated Cohort-Selection (MACS). MACS is a patent-pending technique that combines humans with machines to build large research cohorts without sacrificing data quality. With MACS, we train machine learning models using data generated from abstractors. These models are then used to filter out patients that are highly unlikely to meet the cohort’s inclusion/exclusion criteria. MACS models are continually updated and monitored to ensure they are recent, unbiased, and highly sensitive. All other patients are sent for manual review of the full patient chart, and only included in the cohort upon confirmation of the selection criteria of interest (eg, metastatic disease, pathology). Additional information on MACS can be found on Flatiron’s Knowledge Center.
