# Supplementary material for: Comparative effectiveness of first-line palbociclib plus letrozole versus letrozole alone for HR+/HER2− metastatic breast cancer in US real-world clinical practice
Source: Breast Cancer Res. 2021 Mar 24;23:37. doi: 10.1186/s13058-021-01409-8 (PMC7989035; doi:10.1186/s13058-021-01409-8)
Supplement: Supplementary file 2 — Additional file 2. Patient Characteristics After PSM. [file 13058_2021_1409_MOESM2_ESM.docx]

Additional File 2: Patient Characteristics After PSM

|  | **Cohort After PSM** | | |
| --- | --- | --- | --- |
| Characteristic | Palbociclib + Letrozole  (n=464) | Letrozole  (n=464) | Standardized Difference |
| Age, y |  |  |  |
| Mean (SD) | 66.5 (10.9) | 67.0 (10.9) | –0.0449 |
| Median (IQR) | 68 (59.0–75.0) | 67 (60.0–76.0) |  |
| Age group,* n (%), y |  |  |  |
| 18−49 | 33 (7.1) | 27 (5.8) | 0.0526 |
| 50–64 | 151 (32.5) | 163 (35.1) | –0.0547 |
| 65–74 | 161 (34.7) | 152 (32.8) | 0.0410 |
| ≥75 | 119 (25.6) | 122 (26.3) | –0.0147 |
| Race/ethnicity,* n (%) |  |  |  |
| White | 319 (68.8) | 318 (68.5) | 0.0046 |
| Black | 37 (8.0) | 40 (8.6) | –0.0234 |
| Asian | 5 (1.1) | 6 (1.3) | –0.0199 |
| Hispanic or Latino | 12 (2.6) | 11 (2.4) | 0.0139 |
| Not documented^†^ | 91 (19.6) | 89 (19.2) | 0.0109 |
| Practice type,* n (%) |  |  |  |
| Academic | 29 (6.3) | 20 (4.3) | 0.0868 |
| Community | 435 (93.8) | 444 (95.7) |  |
| Disease stage at initial diagnosis,* n (%) |  |  |  |
| I | 55 (11.9) | 51 (11.0) | 0.0271 |
| II | 105 (22.6) | 102 (22.0) | 0.0155 |
| III | 61 (13.1) | 66 (14.2) | –0.0314 |
| IV | 197 (42.5) | 192 (41.4) | 0.0218 |
| Not documented | 46 (9.9) | 53 (11.4) | –0.0489 |
| ECOG PS,* n (%) |  |  |  |
| 0 | 144 (31.0) | 135 (29.1) | 0.0423 |
| 1 | 92 (19.8) | 108 (23.3) | –0.0839 |
| 2, 3, or 4 | 38 (8.2) | 38 (8.2) | 0.0000 |
| Not documented | 190 (40.9) | 183 (39.4) | 0.0308 |
| Visceral disease,* ^‡^ n (%) |  |  |  |
| No | 298 (64.2) | 285 (61.4) |  |
| Yes | 166 (35.8) | 179 (38.6) | –0.0580 |
| Bone-only disease,*^§^ n (%) |  |  |  |
| No | 272 (58.6) | 276 (59.5) |  |
| Yes | 192 (41.4) | 188 (40.5) | 0.0175 |
| Brain metastases, n (%) |  |  |  |
| No | 453 (97.6) | 440 (94.8) |  |
| Yes | 11 (2.4) | 24 (5.2) | –0.1475 |
| Time from initial Dx to metastatic Dx,* n (%), y |  |  |  |
| De novo | 197 (42.5) | 192 (41.4) | 0.0218 |
| ≤1 | 12 (2.6) | 12 (2.6) | 0.0000 |
| >1–5 | 67 (14.4) | 76 (16.4) | –0.0537 |
| >5 | 188 (40.5) | 184 (39.7) | 0.0176 |
| Not documented | 0 | 0 | NA |
| Number of metastatic sites,*^ǁ^ n (%) |  |  |  |
| 1 | 260 (56.0) | 256 (55.2) | 0.0174 |
| 2 | 120 (25.9) | 122 (26.3) | –0.0098 |
| 3 | 48 (10.3) | 51 (11.0) | –0.0209 |
| 4 | 17 (3.7) | 18 (3.9) | –0.0113 |
| ≥5 | 9 (1.9) | 9 (1.9) | 0.0000 |
| Not documented | 10 (2.2) | 8 (1.7) | 0.0313 |

Dx=diagnosis; ECOG PS=Eastern Cooperative Oncology Group performance status; IQR=interquartile range; NA=not available; PSM=propensity score matching.

*Variable used in PSM model; de novo vs not de novo were used as categories for initial Dx to metastatic Dx.

^†^Race data were not known in the “not documented” race group.

^‡^Visceral disease was defined as metastatic disease in the lung and/or liver; patients could have had other sites of metastases. No visceral disease was defined as no lung or liver metastases.

^§^Bone-only disease was defined as metastatic disease in the bone only.

^ǁ^Multiple metastases at the same site were counted as 1 site (eg, if a patient had 3 bone metastases in the spine, it was considered only 1 site).

The balance in important prognostic baseline characteristics was assessed using a standardized differences approach, with a standardized difference of ≥0.10 considered indicative of practical significance [24].
