# Supplementary material for: Comparative effectiveness of first-line palbociclib plus letrozole versus letrozole alone for HR+/HER2− metastatic breast cancer in US real-world clinical practice
Source: Breast Cancer Res. 2021 Mar 24;23:37. doi: 10.1186/s13058-021-01409-8 (PMC7989035; doi:10.1186/s13058-021-01409-8)
Supplement: Supplementary file 4 — Additional file 4. Forest Plot of Overall Survival by Subgroup* After PSM. [file 13058_2021_1409_MOESM4_ESM.docx]

**Additional File 4**. Forest Plot of Overall Survival by Subgroup* After PSM


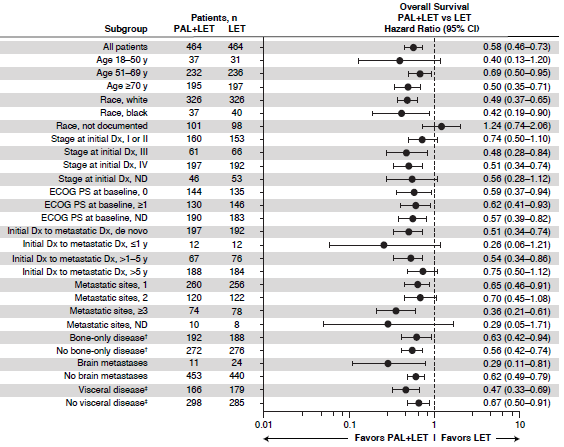


Dx=diagnosis; ECOG PS=Eastern Cooperative Oncology Group performance status; LET=letrozole; ND=not documented; PAL=palbociclib; PSM=propensity score matching.

*Race by Cohort interaction was the only subgroup variable-by-treatment cohort interaction that was significant (*P*=0.0076); however, race data were not known in the “not documented” race group.

^†^Bone-only disease was defined as metastatic disease in the bone only.

^‡^Visceral disease was defined as metastatic disease in the lung and/or liver; patients could have had other sites of metastases. No visceral disease was defined as no lung or liver metastases.
